# Nutritional Factors, Physical Health and Immigrant Status Are Associated with Anxiety Disorders among Middle-Aged and Older Adults: Findings from Baseline Data of The Canadian Longitudinal Study on Aging (CLSA)

**DOI:** 10.3390/ijerph17051493

**Published:** 2020-02-26

**Authors:** Karen M. Davison, Shen (Lamson) Lin, Hongmei Tong, Karen M. Kobayashi, Jose G. Mora-Almanza, Esme Fuller-Thomson

**Affiliations:** 1 Health Science, Kwantlen Polytechnic University, Surrey, BC V3W 2M8, Canada; jgerardomoral@gmail.com; 2 Department of Psychology, University of Hawai’i at Mānoa, Honolulu, HI 96822, USA; 3 Factor-Inwentash Faculty of Social Work and Institute for Life Course & Aging, University of Toronto, Toronto, ON M5S 1V4, Canadaesme.fuller.thomson@utoronto.ca (E.F.-T.); 4 Faculty of Health and Community Studies, MacEwan University; Edmonton, AB T5J 4S2, Canada; tongh8@macewan.ca; 5 Department of Sociology, University of Victoria; Victoria, BC V8W 2Y2, Canada; kmkobay@uvic.ca; 6 Department of Family & Community Medicine and Faculty of Nursing, University of Toronto, Toronto, ON M5G 1V7 & M5T 1P8, Canada

**Keywords:** nutrition, immigration, determinants of mental health, CLSA, anxiety disorders

## Abstract

The main purpose of this study was to compare the lifetime prevalence of anxiety disorders among foreign-born and Canadian-born adults in middle and later life. Using baseline data of the Canadian Longitudinal Study on Aging (2010–2015), multivariable binary logistic regression was conducted to investigate anxiety diagnosis and immigrant status, while controlling for socio-economic, health-related, and nutrition covariates. Of 26,991 participants (49.3% men, 82.5% Canadian born, 58.5% aged 45–65 years), the overall prevalence of self-reported physician diagnosis of anxiety disorders was 8.5%, with immigrants being lower than Canadian-born respondents (6.4% vs. 9.3%, *p* < 0.001). After accounting for all covariates, the adjusted odds ratio (aOR) for anxiety disorders was lower among immigrants (aOR = 0.77, 95% CI: 0.67–0.88) compared to those who were Canadian born. Identified risk factors included: younger age (aORs = 1.79–3.52), being a woman (aOR = 1.25, 95% CI: 1.07–1.46), single status (aOR = 1.27, 95% CI: 1.09–1.48), lower income (aORs = 1.28–2.68), multi-morbidities (aORs = 2.73–5.13), chronic pain (aOR = 1.31, 95% CI: 1.18–1.44), lifetime smoking ≥ 100 cigarettes (aOR = 1.35, 95% CI: 1.23–1.48), BMI < 18.5 (aOR = 1.87, 95% CI: 1.20–2.92), body fat ≥ 26% (aORs = 1.28–1.79), fruit and vegetable intake (<3/day; aORs = 1.24–1.26), and pastry consumption (>1/day; aOR = 1.55, 95% CI: 1.12–1.15) (*p* < 0.05). Targeting socio-economic and nutritional risk factors may reduce the burden of anxiety disorders in middle and late adulthood.

## 1. Introduction

Approximately one-tenth of the global population will suffer from an anxiety disorder [1,2,3]. Based upon assessment of years of life lived with disabilities, anxiety disorders are ranked within the top ten causes of disability in most global regions [4]. Those with anxiety disorders have lower satisfaction with intimate relationships [5], lower well-being [6], as well as increased risk of suicide ideation and attempts [7,8,9] compared to those without anxiety disorders. In Canada, the costs of lost productivity due to anxiety are estimated at $17.3 billion annually [10,11]. In the United States, the annual direct medical costs associated with anxiety disorders are estimated at $33.71 billion [12,13].

The occurrence of anxiety disorders varies across different demographic, socio-economic, and health-related factors. Anxiety disorders have been reported to be twice as common in women compared to men [14]. They are more common among younger adults than older adults [15], and those with lower income [16], and/or less education [17]. Men and women who are in a first marriage have a lower likelihood of developing an anxiety disorder when compared to those who have always been single or who had been previously married [18]. Various health determinants are also associated with lower or higher levels of anxiety disorders. Adults who are obese [19] or who have chronic conditions such as migraines [20], inflammatory bowel disease [21], or cancer [22,23] are more likely to have anxiety disorders compared to those without these conditions. Health behaviors, such as regular exercise, are associated with lower levels of anxiety [24,25,26]. Conversely, those diagnosed with anxiety are more likely to engage in unhealthy behaviors such as smoking and heavy drinking [19]. Different dietary factors may contribute to heightened or lowered levels of anxiety disorders. For example, women who followed a “Western” diet high in refined grains, sugary products, and processed foods were found to be significantly more likely to have an anxiety disorder compared to those with a balanced diet consisting of fruits, vegetables, meat, fish, and whole grains [27,28]. Additionally, higher intakes of omega-3 fatty acids [29], nuts [30], fiber [31,32], and lower intakes of salt [33] are associated with lower likelihood of an anxiety disorder.

Recent research with the Canadian Longitudinal Study on Aging (CLSA) indicates that immigrants, particularly women, are vulnerable to mental health conditions such as depression and psychological distress [34,35]. This may be due to the fact that immigrants face substantial life challenges when they age in a foreign land, such as devaluation of qualifications [36], downward shifts in social mobility, racial discrimination [37,38], dietary transition, and food insecurity [39]—all of which could potentially trigger psychological dysfunction. Both social class and social mobility have been associated with psychiatric disorders and need to be accounted for in any analyses of mental health outcomes [40].

The literature about the relationships between immigrant status and mental health conditions such as anxiety disorders is paradoxical. Despite the above-mentioned post-immigration stressors, recent studies have shown that immigrants tend to report lower rates of anxiety disorders compared to those born in the host country [41,42]. For instance, compared to those born in the U.S., immigrants have a much lower prevalence of anxiety within specific racial/ethnic groups [41], such as Africans (6.0% vs. 13.1%), Asian Americans (6.6% vs. 16.2%), European Americans (10.5% vs. 13%), and Mexican American (8.5% vs. 12.6%). Similar findings have been documented in Canada where the Canadian born were twice as likely to be diagnosed with generalized anxiety disorder compared to immigrants [43]. This “healthy migrant advantage” may be attributable to ‘selection effects’, whereby those with physical health conditions or mental health problems, such as anxiety disorders, may be less likely to immigrate [44]. Conversely, those who choose to immigrate may engage in health behaviors that are protective against poor mental health [45]. 

Health care professionals and many other stakeholders have highlighted the need to identify contributing factors to mental health disparities as public health priorities, especially among marginalized populations [45]. In Canada, later-life mental health problems remain understudied among immigrant populations [46]. Thus, this study analyzed baseline data from the CLSA to investigate the prevalence and modifiable risk factors related to anxiety disorders in middle-age and older Canadian-born and immigrant adults. It aims to provide direction for evidence-based programming and policy in mental health screening, diagnoses, and treatments to help reduce the burden of anxiety disorders among these targeted populations. Informed by previous scientific evidence centering on a social determinants of mental health framework, this study was guided by the following four research questions:

(1) Is immigrant status associated with anxiety disorders among Canadians aged 45 to 85?
(2) Is the association between immigrant status and anxiety disorders attenuated by a wide range of socio-demographic, health, and nutritional correlates?
(3) What specific dietary intakes are associated with anxiety disorders among Canadians 45–85 years after adjusting for immigrant status?
(4) What other factors are significantly associated with anxiety disorders after controlling for immigrant status?

## 2. Materials and Methods

### 2.1. Sample

The complete methodology has been described in detail in earlier articles [34,35]. This study analyzed the baseline (2012–2015) comprehensive cohort of the CLSA (www.clsa-elcv.ca). Briefly, the CLSA is an ongoing nationwide cohort study of 50,000 Canadian residents aged 45–85 years using a complex sampling frame. In the comprehensive cohort, 30,097 participants completed in-home interviews at baseline, where social and health-related information was collected [47]. In addition, they underwent physical assessment and provided biospecimen samples at specific study sites [48]. Those with no response (don’t know or refused) to the question of anxiety disorders were excluded, yielding a final sample size of 26,991. Data collection sites included Vancouver, Surrey, Victoria, Calgary, Winnipeg, Hamilton, Ottawa, Montreal, Sherbrooke, Halifax, and St. John’s. Excluded from the sample are individuals without a residential address, residents of Canada’s territories and remote regions, First Nations living on reserves, members of the Canadian Armed Forces, individuals living in long-term care institutions, temporary visa holders, individuals unable to speak English or French, and individuals with cognitive impairment. The CLSA study protocol has been approved by 13 research ethics boards across Canada. Approval to conduct this secondary analysis of CLSA data was granted by the University of Toronto’s Health Sciences Human Participant Ethics Review Board (Protocol number: 34065). The data is only accessible to select investigators who have permission to analyze it. 

### 2.2. Measures

The dependent variable, self-reported lifetime anxiety disorder, was based on an affirmative response to the yes/no question “Has a doctor ever told you that you have an anxiety disorder such as a phobia, obsessive-compulsive disorder or a panic disorder?”. The prevalence of lifetime diagnosis of anxiety disorder was calculated as the proportion of participants who chose “yes”. A similar question was asked in Statistics Canada’s Canadian Community Health Survey and has been applied in other peer-reviewed studies [49,50,51]. Past research has suggested that a self-reported medical diagnosis of mental health outcome is valid in population-based studies [52,53]. 

The independent variable of interest, immigrant status (dichotomized as Canadian-born resident versus Canadian immigrant), was derived from the question that asked about country of birth. A broad range of demographic, social, economic, health, and nutrition-related covariates that could potentially attenuate the relationship between anxiety disorders and immigrant status were also examined [45,46]. The definition and operationalization of covariates are outlined in supplementary Table S1.

### 2.3. Analysis

All analyses were completed using SPSS Version 25 (IBM Corp, Armonk, NY: IBM Corp.). Unweighted counts were used to describe the entire sample itself, and weighted percentages were used to describe the study population stratified by prevalence of anxiety disorders and immigration status. This rescaled version of the full population survey weights was applied in the analysis to represent the total population of Canadians between 45 and 85 years. It was calculated by dividing the CLSA’s trimmed inflation weight used in this study sample by the average unit of the full survey weights. Because those with missing data may be more likely to experience an anxiety disorder, many variables in the analysis retain the “no answer” as a category in the analyses to avoid potential underestimation.

Binary logistic regression was used due to the cross-sectional study design and the dichotomous categorization of anxiety disorder as the outcome variable. This multivariable analysis provided adjusted odds ratios (ORs) and 95% confidence intervals (95% CI) of the primary relationship of interest, the association between immigrant status and anxiety disorders, while adjusting for the covariates. Six stepwise models were analyzed to explore the attenuation of the immigrant-anxiety disorder relationship based upon 7 distinct clusters of variables. Model 1 (age, sex and immigration status) was the baseline model. Model 2 included socio-economic variables in addition to Model 1. Model 3 included physical health characteristics in addition to Model 1. Model 4 included health behavior variables in addition to Model 1. Model 5 included indicators of over-nutrition in addition to Model 1 and Model 6 included dietary intake measures in addition to Model 1. Model 7 included all the variables. Each model included clusters of factors that have been previously reported to be associated with mental health outcomes.

A sensitivity analysis was performed because visible minority middle-aged and older adults in Canada are much more likely to be immigrants than Canadian born, and ethnicity status and immigration status may be confounded in the sample. The sensitivity analysis examined if visible minority status was an important contributor to the relationship between immigrant status and anxiety disorders. The fully adjusted logistic regression analysis (Model 7) was replicated with a 4-level immigrant status/visible minority variable (visible minority immigrant, white immigrant, visible minority Canadian born, and White Canadian born) replacing the dichotomous immigrant status variable (Immigrant vs. Canadian born). Participants who were white and born in Canada were the reference category. 

## 3. Results

### 3.1. Sample Description and Bivariate Analysis

Among 26,991 participants (see Table 1), the majority were Canadian-born residents (82.5%), 45–65 years (58.5%), earning between C$20,000 and $99,999/year (53.9%), in a relationship (69.6%), and who had earned a post-secondary diploma or degree (78.2%). The sample is almost equally distributed by sex (men: 49.3%; women: 50.7%). Among immigrants (n = 4733), the average time since arrival in Canada was 43 years (SD ± 15.8 years). Fewer than 1% of immigrants (n = 193) had arrived in Canada in the 10 years preceding the baseline CLSA survey and 37.6% (n = 1779) had arrived 50 or more years before they were surveyed. In terms of racial status, the sample was predominately Canadian-born whites (81.6%, n = 22,037), followed by 13.8% foreign-born whites (n = 3736), 3.4% foreign-born visible minorities (n = 914), and 1% Canadian-born visible minorities (n = 275). 

Based on physical health and health behavior indicators, more than 80% had at least one health condition (81.7%) with almost one-third having some stage of hypertension (26.4%) and another one-third taking anti-hypertensive medication (27.9%). Most of the participants did not report having chronic pain (78.8%), did not binge drink (64.7%), and, in the seven days prior to data collection, never or seldom engaged in light sports or recreational activities (89.3%). Slightly more than one-half of the sample had smoked more than 100 cigarettes in their lifetime (52.9%). Based on the anthropometric and dietary intake measures, most respondents had BMIs (BMIs ≥ 25, 69.4%) and waist-to-hip ratios (65.6%) that exceeded the healthy ranges; and disease risk measures suggested that most were at some degree of risk for developing chronic conditions (70.6%). Finally, most respondents consumed low amounts of fiber (≤2 servings/day; 79.4%), pulses and nuts (<1 serving/day; 54.9%), and fruits and vegetables (≤4 servings/day; 63.0%).

In the sample, 2286 out of 26,991 Canadians had been diagnosed with an anxiety disorder, resulting in a total prevalence estimate of 8.5%. Among those born in Canada, 9.3% reported that they had been diagnosed with an anxiety disorder by a health professional at some point during their lifetime. The prevalence estimate of anxiety was 6.4% among immigrants. Significant associations were found between self-report of physician-diagnosed anxiety disorder and all demographic, social, and economic factors (*p* < 0.001), most physical health and health behavior variables (*p* < 0.001) except hypertension and physical activity, and most anthropometric measures (*p* < 0.001) except waist-to-hip ratio. One-half of the dietary intake measures showed significant association with anxiety disorder status (*p* < 0.05); intakes of fiber sources, omega 3 eggs, fruit juice, salty snacks, calcium sources with vitamin D, and chocolate bars were not significantly associated with anxiety disorders.

### 3.2. Logistic Regression Analysis

#### 3.2.1. Research Question 1 & 2: Is Immigrant Status Associated with Anxiety Disorders and Is This Association Attenuated by a Wide Range of Health Determinants?

**Figure 1:**
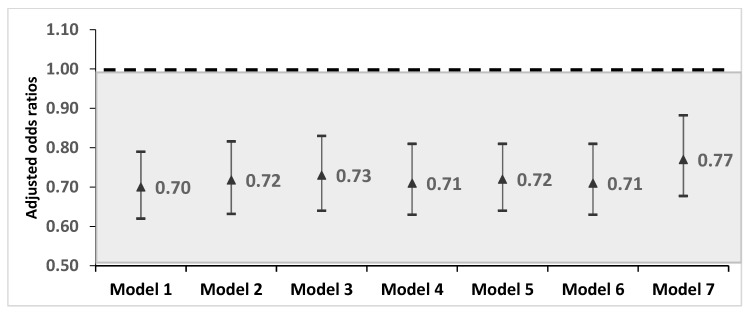
Adjusted odds ratios and 95% confidence intervals for anxiety disorders among immigrants (n = 4733) compared to Canadian-born respondents (n = 22,258). Model 1 (Core)—Demographics: Immigrant status, age, sex; Model 2 (Social and Economic Characteristics)—Model 1 plus: Income, marital status, education; Model 3 (Physical Health)—Model 1 plus: Co-morbidities, hypertension, chronic pain; Model 4 (Health Behaviors)—Model 1 plus: smoking, binge drinking, physical activity, family physician visits; Model 5 (Anthropometric Measures)—Model 1 plus: Disease risk, percent body fat, waist to hip ratio, waist to height ratio; Model 6 (Dietary Intakes)—Model 1 plus: Fiber, pulses/nuts, fat sources, fish sources, omega 3 eggs, fruits and vegetables, pure fruit juice, salty snacks, calcium sources with high vitamin D content, calcium sources with low vitamin D content, pastries, chocolate bars; Model 7 (Complete)—Adjusted for all aforementioned variables.

The age-sex-adjusted odds of anxiety disorders among immigrants were significantly lower (OR = 0.70; 95% CI 0.62–0.79) than in the Canadian-born residents. As shown in Figure 1 (Model 1 to Model 7), the association between immigrant status and anxiety disorders was only modestly attenuated by six clusters of risk factors (ORs 0.70–0.77). After 29 potential risk factors were taken into account, the adjusted odds ratio (aOR) of anxiety disorders among immigrants was 0.77 (95% CI: 0.67–0.88) compared to their Canadian-born counterparts, suggesting that the association was robust.

#### 3.2.2. Research Question 3: What Specific Dietary Intakes Are Associated with Anxiety Disorders after Adjusting for Immigrant Status?

Those who consumed a daily average of two or more fiber sources had higher odds of an anxiety disorder (ORs 1.25–1.34, *p* < 0.05), whereas those who consumed between 0.5 and two sources of pulses and nuts had lower odds of anxiety disorder (ORs 0.84–0.98, *p* < 0.05). Compared to those with high intakes of fruits and vegetables, those who ate less than three sources per day had an increased odds of anxiety disorders (ORs 1.24–1.26, *p* < 0.05). Those with low intakes of calcium sources with high vitamin D content had lower odds of an anxiety disorder (OR = 0.73, *p* < 0.05). Finally, those with average daily intakes of one or more pastries had higher odds of an anxiety disorder (OR = 1.55, 95% CI 1.12–1.15, *p* < 0.05).

#### 3.2.3. Research Question 4: What Other Factors Are Significantly Associated with Anxiety Disorders after Controlling for Immigrant Status?

Significant associations were observed in terms of socio-demographic and health-related variables (see Table 2). Women had 25% higher odds of an anxiety disorder compared to men (aOR = 1.25, 95% CI 1.07–1.46) and those less than 76 years old were about 2 to 3.5 times more likely to have an anxiety disorder compared to those aged 76 to 85 years (aORs = 1.79–3.52, *p* < 0.001). Compared to those earning more than C$150,000/year, the odds of an anxiety disorder increased as annual incomes decreased (aORs = 1.28-2.68, *p* < 0.05), suggesting an inverse relationship between income and anxiety disorders. Compared to adults who were married, those who were single had 27% higher odds of anxiety disorders (aOR = 1.27, 95% CI: 1.09–1.48). 

Among health-related measures, individuals with at least one health condition (aORs = 2.13–5.73, *p* < 0.001) and those who were in chronic pain (aOR = 1.31, 95% CI 1.18–1.44, *p* < 0.001) had higher odds of anxiety disorders compared to those with no health conditions or chronic pain. Among health behaviors, those who smoked 100 or more cigarettes (aOR = 1.35, 95% CI 1.23–1.48, *p* < 0.001) had higher odds of an anxiety disorder compared to those who smoked less than 100 cigarettes over their lifetime. Lower odds of an anxiety disorder were also indicated for those who reported occasional binge drinking (aOR = 0.84, 95% CI 0.75–0.94, *p* = 0.003) compared to those who did not binge drink. Similarly, those who did not visit a physician in the past year had lower odds of anxiety disorders (aOR = 0.85, 95% CI 0.73–0.99, *p* = 0.039) compared to those who had at least one visit. Those with BMIs < 18.5 (underweight) were approximately two times more likely to have anxiety disorders (aOR = 1.87, 95% CI: 1.20–2.92), compared to those with BMI of normal weight (aORs = 0.34–0.53, *p* < 0.05). Those with a higher proportion of body fat (i.e., ≥26%) had higher odds of anxiety disorders (aORs = 1.28–1.79, *p* < 0.05) than their leaner peers.

### 3.3. Sensitivity Analysis

A sensitivity analysis was conducted to determine whether visible minority status, rather than immigration status, was the underlying reason for the significant observed association with anxiety. In the fully adjusted analysis (not shown), the odds of anxiety disorders did not differ significantly for visible minority respondents born in Canada (*p* = 0.29) when compared to whites born in Canada. However, there were significant differences for both white immigrants and non-white immigrants, when compared to Canadian-born whites: White immigrants had 17% lower odds of anxiety disorders (aOR = 0.83, 95% CI 0.72-0.95) and visible minority immigrants had 46% lower odds of anxiety disorders (aOR = 0.54, 95% CI 0.39-0.74) than whites born in Canada. 

## 4. Discussion

Based on a very large sample of middle-aged and older Canadian adults between 45 and 85 years of age, there were lower odds of lifetime physician-diagnosed anxiety disorders among immigrants compared to their Canadian-born participants. This finding was consistent after adjusting for a wide range of demographic, social, economic, health and nutritional factors. This study also examined a wide range of other factors and their relationships with anxiety disorders among middle-aged and older Canadians, including immigrants, which helps to provide information for health-care related decision-making.

### 4.1. Research Questions 1 & 2: Is Immigrant Status Associated with Anxiety Disorders and Is Any Immigration-Anxiety Association Attenuated When Other Health Determinants Are Taken into Account?

Consistent with previous findings [50], the results of this study found that immigrants living in Canada had 30% lower age-sex-adjusted odds of lifetime physician-diagnosed anxiety disorders. This relationship was only modestly altered, to 23% lower odds, when a wide range of demographic, social, economic, physical health, health behavior, nutrition status, and dietary intake factors were considered in the analysis. One possible explanation for this “healthy immigrant effect” may relate to Canada’s rigorous “point system” which gives preference to those with graduate degrees and excellent job prospects [42]. Immigrants with these characteristics may be less vulnerable to anxiety disorders. Another potential explanation for the “healthy immigrant effect” with respect to anxiety is that potential immigrants with anxiety disorders would find the challenges of relocation too anxiety inducing and would therefore not choose to immigrate. If immigrants face barriers to mental health services, the lack of these services may contribute to the lower rate of self-reported diagnosis by a health professional of mental health problems among immigrants [55]. Previous studies have also shown that immigrants are less likely to access mental health services [56] compared to non-immigrants. A scoping review about mental health access in Canada indicated that language barriers between mental health practitioners and immigrants result in underuse of needed mental health care services, and that mental health problems among immigrants are exacerbated when health care practitioners are unable to empathize or understand the needs of these immigrants due to the difference in cultural backgrounds [57]. Similarly, in a systematic review of immigrant access to mental health services in the US, it was also found that stigma was a major barrier in accessing mental health services for most immigrants [58]. To address this concern, we adjusted for visits in the past year with a physician (in Model 4 of Figure 1) and found that physician visits, in conjunction with the health behaviors of smoking, binge drinking, and level of physical activity, did not attenuate the association between immigrant status and lower odds of anxiety disorders.

### 4.2. Research Question 3: What Specific Dietary Intakes Are Associated with Anxiety Disorders after Adjusting for Immigrant Status?

The findings indicating lower odds of anxiety disorders for those with higher intakes of fruits and vegetables, as well as pulses and nuts, are consistent with the results of other studies [28,29,30]. Nuts may be protective against anxiety disorders because certain types, such as walnuts, are good sources of omega-3 fatty acids. In turn, omega-3 fatty acid intake is associated with reduced symptoms of anxiety [59,60]. Furthermore, diets rich in nuts and pulses may reduce inflammatory markers [61] related to anxiety. Diets rich in fruits, vegetables, nuts, and pulses provide a broad range of essential nutrients such as zinc [62], magnesium [63], and B vitamins [64], which have been associated with decreased anxiety.

The findings indicating heightened odds of anxiety disorder with intakes of pastries are also consistent with the results of other studies [28,29]. Some investigations suggest that individuals with anxiety may self-pacify with sugary products as “comfort food” [65]. Alternatively, high intakes of sweets may disrupt the microbiome, result in bacterial translocation, induce inflammatory pathways [66], and promote increases in anxiety levels [67].

The association found between higher odds of anxiety disorder and greater consumption of fiber sources contradicts previous studies which indicate that high fiber intake may be protective against anxiety disorders [31,32]. The phytates in high fiber foods which bind with micronutrients such as iron, zinc, and manganese and lessen their bioavailability, may help to explain the higher odds of anxiety disorders with increased fiber sources [68,69]. The question in the CLSA about fiber intakes focuses on insoluble fiber types. Other studies suggest that it is the soluble types of fiber that may have more beneficial impact on the gut microbiota and mental health [70,71]. Furthermore, the question is specific to high fiber grain products and does not necessarily consider fat sources (e.g., butter on whole grain bread) which may be added to these foods.

To the best of our knowledge, the finding that low intakes of calcium sources with high vitamin D content are associated with lower odds of anxiety disorders has not been reported elsewhere. Interestingly, the lower odds ratios trended towards the null value as the number of sources increases, suggesting a possible dose response effect. It is also important to note that supplemental sources of calcium and vitamin D, that are recommended and commonly taken by older adults, were not accounted for in this analysis. In addition, biochemical indicators of vitamin D were not analyzed. Further research would be needed to assess vitamin D status and its potential association with anxiety disorders.

### 4.3. Research Question 4: What Other Factors Are Significantly Associated with Anxiety Disorders after Controlling for Immigrant Status?

Consistent with other studies, there was a higher likelihood of anxiety disorders in women [14], younger respondents [16], individuals with lower income [17], and who had never married [19]. The findings of higher odds of anxiety disorders among those with chronic health conditions and pain have been reported in earlier studies [21,23,24]. The higher odds of anxiety disorders may be attributed to the mental health consequences of experiencing physical health problems. Other factors may be due to central nervous system effects and peripheral inflammatory changes that can cause changes in central nervous system sensitization and lead to heightened reactivity that may worsen affective distress [72].

Consistent with previous investigations, those who smoked [20], were obese [20], and visited physicians annually [73] were more likely to report a lifetime diagnosis of anxiety disorders. There are many potential mechanisms which link obesity and anxiety. Being obese results in a cascade of negative social interactions due to stigma and thus, weight discrimination may cause anxiety in some individuals who are obese [74]. Obesity is also linked to functional impairments [75] and a variety of chronic illnesses which increase the risk of having anxiety disorders. Researchers have suggested that anxiety disorders impair the regular functions of the hypothalamic-pituitary-adrenal (HPA) axis, causing an increase in appetite [76] due to alterations in the two hormones, ghrelin and leptin. Symptoms of anxiety have been reported to be negatively associated with levels of leptin [77,78], while ghrelin was found to have an anxiolytic effect in animal studies [79,80] following induced stress. Clearly, further research is needed to better understand the pathways that link obesity and anxiety.

The findings that occasional binge drinking is associated with lower odds of anxiety disorders contradict some previous research [20]. However, other investigations suggest that anxiety is highly associated with heavy drinking [80], defined as more than 14 drinks per week.

### 4.4. Limitations and New Contributions to The Research Literature

Several methodological limitations deserve attention. First, the use of cross-sectional data does not allow causal inferences to be made. Longitudinal analyses and qualitative investigation would provide further insights about causal mechanisms. Second, the question about anxiety disorders included both panic disorder and obsessive-compulsive disorder, which in the most recent psychiatric diagnostic criteria are differentiated. Third, potential subgroup differences by time since immigration could not be examined due to the modest sample size of recent newcomers (i.e., fewer than 1% arrived in the 10 years preceding the survey). Fourth, the self-report outcome measure was not based on a standard screening instrument for measuring current anxiety symptoms. Self-report of a doctor’s diagnosis of anxiety disorders may underestimate the actual prevalence of mental health burden in the sample due to recall bias among older populations [81] or lack of assessment by their doctor. To examine this problem, health care utilization (i.e., physician visits in the past 12 months) was included in the study’s analysis and it was not found to attenuate the lower odds of anxiety disorders among immigrants. However, it is important to note that information about the respondents’ lifetime health care utilization was not available. If immigrants had many fewer visits to health professionals across their life course, they would have less opportunities to be diagnosed with an anxiety disorder. Future investigations could explore this by linking participant data with medical records and by using standardized measures such as the Anxiety Sensitivity Index [82,83]. Finally, although mood disorder was accounted for in the multi-morbidity measurement, depression as measured by a standardized scale was not included in the analysis. As noted by others, depression could be a confounder between anxiety disorders and the various factors that were examined such as female gender [84], chronic pain [85], multimorbidity [86,87], obesity [88], and smoking [89]. Despite being cross-sectional, this population-based study was able to compare immigrants relative to those who were Canadian born in terms of anxiety prevalence by exploring detailed information about nutrition status, dietary intake, and physical assessment, thus providing a valuable contribution to the immigrant health literature.

## 5. Conclusions

Our findings indicate that one in 15 middle-aged and older immigrants have a lifetime diagnosis of anxiety disorders in Canada. The findings highlight that the association between immigration and anxiety disorders is long lasting as more than one-third of immigrants had been in Canada for more than half a century. Although this study found that the odds of anxiety disorders were 33% lower among immigrants than non-immigrants, it remains important to provide appropriate mental health interventions to all Canadians with anxiety disorders. The identified social, health, and nutritional factors associated with anxiety disorders provide helpful information for improving targeted outreach and treatment for mid-age and older adults. Dietary factors, such as fiber, calcium, and vitamin D intakes warrant further study in order to better understand their possible relationships with anxiety disorders. In particular, supplemental sources and biochemical indicators of vitamin D status should be accounted for. Promising evidence-based psychological interventions for anxiety, such as cognitive-behavioral therapy [90], may help to minimize the burden of anxiety disorders among all middle-aged and older adults, both immigrants and those born in Canada.

## Figures and Tables

**Table 1:** Prevalence estimates of immigration status and anxiety diagnosis by sample characteristics.

Variables	Total (n = 26,991)		Immigration Status		Cases of Anxiety		X^2^ (df), *p*-Value ^a^
			CB	FB	Disorders		
			(n = 22,258)	(n = 4733)	(n = 2286)		
	n	%	CB%	FB%	n	Anxiety%	
Core Block							
Immigration status							
Canadian born (CB)	22,258	82.8%	--	--	2007	9.3%	40.8 (1), <0.001
Foreign born (FB)	4733	17.2%	--	--	279	6.4%	
Sex							
Men	13,300	49.9%	49.1%	53.9%	849	6.7%	153.5 (1),
Women	13,691	50.1%	50.9%	46.1%	1437	11.0%	<0.001
Age							
45–55 years	6862	42.3%	42.9%	39.1%	666	9.2%	56.2 (3), <0.001
56–65 years	8947	30.0%	30.9%	25.6%	864	10.0%	
66–75 years	6628	17.1%	16.1%	22.0%	523	8.0%	
76–85 years	4554	10.7%	10.1%	13.3%	233	5.6%	
Socio-Demographic Factors							
Income							
<$20,000	1237	3.9%	4.1%	3.2%	247	22.0%	312.8 (5), <0.001
$20,000–$49,999	5514	17.1%	16.9%	18.0%	523	10.1%	
$50,000–$99,999	9042	31.9%	31.6%	33.1%	760	9.3%	
$100,000–$149,999	5069	21.4%	21.7%	19.8%	372	7.7%	
≥$150,000	4476	20.6%	20.7%	19.8%	258	5.8%	
Not answered	1653	5.2%	5.1%	6.1%	126	9.2%	
Marital status							
Single	2300	3.9%	8.7%	5.0%	288	13.9%	131.1 (2), <0.001
Live with a partner	18,781	17.1%	75.8%	80.6%	1397	7.8%	
Widowed/separated	5910	31.9%	15.5%	14.4%	601	11.4%	
Educational level							
<High school	1378	4.50	5.0%	2.5%	157	12.6%	32.3 (3), <0.001
High school	4471	15.4%	16.0%	12.5%	397	9.2%	
≥Post-secondary	21,099	80.0%	79.0%	84.7%	1726	8.5%	
Non-response	43	0.1%	0.1%	0.3%	6	22.2%	
Physical Health							
Morbidities							
0 health conditions	4946	20.8%	20.5%	22.3%	139	3.0%	829.5 (3), <0.001
1 health condition	7319	28.9%	28.8%	29.7%	414	6.3%	
2 health conditions	6307	22.5%	22.7%	21.7%	484	8.3%	
3 health conditions	8419	27.8%	28.1%	26.30%	1249	16.4%	
Physical Health*/cont’d.*							
Hypertension levels							
Normal	9826	42.5%	42.5%	42.3%	817	8.5%	7.5 (4), 0.11
Elevated	2578	8.7%	8.6%	9.2%	198	8.3%	
Stage 1 hypertension	4303	17.1%	17.4%	15.9%	392	9.2%	
Stage 2 hypertension	2835	9.7%	9.5%	10.5%	240	8.6%	
Takes anti- hypertensives	7449	22.0%	22.0%	22.2%	639	9.6%	
Chronic pain							
No reported pain	21,257	79.8%	79.7%	80.1%	1519	7.5%	252.6 (2), <0.001
Have pain	5629	20.0%	20.10%	19.4%	758	14.3%	
Refused	105	0.2%	0.2%	0.5%	9	9.7%	
Health Behaviors							
Smoking lifetime							
≥100 cigarettes	14,290	50.7%	51.9%	44.8%	1376	10.5%	91.5 (1),
<100 cigarettes	12,701	49.3%	48.1%	55.2%	910	7.2%	<0.001
Binge drinking ^b^							
Non-binge drinking	17,472	60.2%	58.5%	68.4%	1495	9.2%	16.7 (2), <0.001
Occasional	4224	22.2%	23.0%	18.7%	380	9.2%	
Regular	5295	17.6%	18.5%	12.90%	411	7.5%	
Physical activity							
Never or seldom	24,101	89.7%	89.8%	89.5%	2050	8.9%	2.2 (2), 0.326
Sometime or often	2880	10.2%	10.2%	10.4%	235	8.1%	
No answer /refused	10	0.0%	0.0%	0.2%	1	9.1%	
Family physician visits in the past year							
Yes	24,327	87.6%	87.5%	87.9%	2114	9.2%	29.8 (1)
No	2664	12.4%	12.5%	12.1%	172	6.3%	<0.001
Anthropometric Measures							
Body mass index (BMI)						
Underweight: <18.5	185	0.7%	0.7%	0.7%	26	16.8%	43.8 (3), <0.001
Normal: 18.5–24.9	8064	32.1%	31.5%	35.0%	631	8.4%	
Overweight: 25–29.9	10,901	40.1%	39.9%	40.7%	832	8.0%	
Obese: ≥30	7841	27.2%	27.9%	23.7%	797	10.3%	
Anthropometric Measures */cont’d.*							
Waist-to-hip ratio							
Low risk	9290	36.5%	36.5%	36.8%	827	9.2%	2.9 (2),
High risk	17,699	63.5%	63.5%	63.2%	1458	8.6%	0.228
Waist-to-height ratio							
< cut-off	18,102	67.7%	67.2%	70.1%	1399	8.3%	20.7 (1),
≥ cut-off	8889	32.3%	32.8%	29.9%	887	10.0%	<0.001
Disease risk							
Least risk	7942	31.8%	31.2%	34.8%	622	8.5%	51.5 (3), <0.001
Increased	7070	28.0%	27.5%	30.1%	494	7.3%	
High	4659	15.4%	15.7%	13.8%	414	9.6%	
Very high	7320	24.9%	25.6%	21.3%	756	10.6%	
Body fat percent							
0–26%	4793	20.8%	20.2%	23.8%	243	5.4%	219.1 (5), <0.001
26%–31%	5216	19.5%	19.4%	20.1%	352	7.4%	
31%–36%	5248	18.7%	18.5%	20.1%	407	8.0%	
36%–41%	4995	17.7%	18.2%	15.5%	528	11.5%	
41%–59%	5815	19.6%	20.3%	15.9%	671	12.2%	
Dietary Intakes							
Average daily intake of fiber sources							
<1	8655	34.5%	35.1%	31.3%	781	8.9%	3.3 (3), 0.338
≥1 & <2	12,780	46.9%	46.8%	47.4%	1032	8.6%	
≥2 & <3	4440	14.9%	14.5%	16.8%	372	9.3%	
≥3	1116	3.7%	3.6%	4.4%	101	9.8%	
Average daily intake of pulses and nuts							
<0.5	8625	31.3%	32.0%	28.4%	835	10.1%	24.6 (3), <0.001
≥0.5 & <1	6204	23.1%	23.2%	22.6%	466	7.9%	
≥1 & <2	10,353	38.8%	38.4%	40.4%	831	8.4%	
≥2	1809	6.8%	6.4%	8.7%	154	8.7%	
Average daily intake of fat sources							
<2.5	3304	12.1%	11.2%	16.2%	277	9.0%	8.6 (3), 0.035
≥2.5 & <5	10,202	37.7%	37.2%	39.9%	821	8.4%	
≥4 & <5	6587	24.7%	25.1%	22.5%	554	8.6%	
≥5	6898	25.5%	26.4%	21.4%	634	9.6%	
Intake of fish in the past year							
Never	2240	8.2%	8.5%	6.8%	250	11.5%	21.4 (1),
Ever	24,751	91.8%	91.5%	93.2%	2036	8.6%	<0.001
Intake of omega 3 eggs in the past year							
Never	19,916	72.7%	73.3%	69.8%	1696	8.9%	0.1 (1),
Ever	7075	27.3%	26.7%	30.2%	590	8.7%	0.673
Dietary Intakes */cont’d..*							
Average daily intake of fruits and vegetables							
<2	3817	13.1%	13.5%	11.3%	389	10.5%	21.3 (4), <0.001
≥2 & <3	6446	22.7%	22.8%	22.3%	555	9.2%	
≥3 & <4	6741	25.1%	25.0%	25.3%	518	7.8%	
≥4 & <6	7357	27.9%	27.4%	30.0%	610	8.9%	
≥6	2630	11.2%	11.3%	11.1%	214	8.4%	
Average daily intake of pure fruit juice							
No consumption	8725	31.2%	30.7%	33.7%	775	9.1%	3.2 (2), 0.196
≤1	17,680	66.7%	67.2%	64.2%	1455	8.7%	
>1	586	2.1%	2.1%	2.1%	56	10.4%	
Average daily intake of salty snacks							
No consumption	5086	15.9%	14.0%	25.0%	392	8.2%	2.6 (2), 0.264
>0 & ≤1	21,850	83.9%	85.8%	74.8%	1890	9.0%	
>1 & ≤10	55	0.2%	0.2%	0.2%	4	10.9%	
Average daily intake of calcium sources with high vitamin D content							
<1	5945	22.5%	22.2%	23.7%	500	8.8%	19.6 (3), <0.001
≥1 & <2	12,233	45.6%	45.3%	46.9%	983	8.3%	
≥2 & <4	7839	28.4%	28.9%	26.5%	692	9.3%	
≥4	974	3.5%	3.6%	2.9%	111	12.2%	
Average daily intake of calcium sources with low vitamin D content							
No consumption	4480	16.3%	16.9%	13.7%	412	8.6%	0.2 (1),
>0	20,225	83.7%	83.1%	86.3%	1874	8.9%	0.599
Average daily intake of pastries							
No consumption	2733	10.0%	9.5%	12.1%	253	9.5%	10.0 (2), 0.007
>0 & ≤1	23,794	88.3%	88.6%	86.9%	1977	8.7%	
>1	464	1.7%	1.8%	1.0%	56	12.6%	
Average weekly intake of chocolate bars							
No consumption	9354	34.0%	34.7%	30.4%	784	8.9%	2.7 (2), 0.252
>0 & <0.6	16,420	61.7%	61.40%	63.3%	1379	8.7%	
≥0.6	1217	4.3%	3.9%	6.3%	123	10.1%	

^a^ Chi-square tests (X^2^) were performed based on the presence or absence of anxiety disorders by various variables. ^b^ <once/month but ≥ once/past 12 months; regular binge drinking (i.e., men who had ≥ five drinks or women who had ≥ four drinks on one occasion ≥ once/month in the past 12 months) [54]. CB = Canadian born; FB = foreign born.

**Table 2:** Adjusted odds ratios of anxiety disorders by immigrant status, socio-demographic, physical health, health behavior, over- and under-nutrition, and dietary measures (Model 7).

Variable	aOR (95% CI)	*p*-Value
Demographic, Social, and Economic Characteristics		
Immigrant (Ref: Canadian born)		
Immigrant	**0.77 (0.67–0.88)**	**<0.001**
Age (Ref: 76–85 years)		
45–55 years	**3.52 (2.88–4.29)**	**<0.001**
56–65 years	**2.86 (2.36–3.46)**	**<0.001**
66–75 years	**1.79 (1.47–2.18)**	**<0.001**
Sex (Ref: Male)		
Female	**1.25 (1.07–1.46)**	**0.006**
Income (Ref:≥$150,000)		
< $20,000	**2.68 (2.14–3.37)**	**<0.001**
$20,000–49,999	**1.47 (** **1.23–1.74)**	**<0.001**
$50,000–99,999	**1.43 (1.23–1.65)**	**<0.001**
$100,000–149,999	**1.28 (1.09–1.49)**	**0.002**
Not answered	**1.39 (1.10–1.75)**	**0.005**
Marital status (Ref: Married/common law)		
Single	**1.27 (1.09–1.48)**	**0.002**
Widowed/divorced/separated	1.08 (0.95–1.23)	0.255
Education level (Ref: < high school)		
High school graduate	0.83 (0.67–1.03)	0.089
Post-secondary degree/diploma	0.92 (0.75–1.12)	0.394
Not answered	2.22 (0.91–5.43)	0.080
Physical Health		
Morbidities (Ref: No health conditions)		
1 health condition	**2.13 (1.78–2.56)**	**<0.001**
2 health conditions	**2.79 (2.33–3.35)**	**<0.001**
3 health conditions	**5.73 (4.81–6.82)**	**<0.001**
Hypertension levels (Ref: Normal blood pressure)		
Elevated	1.02 (0.86–1.21)	0.823
Stage 1 hypertension	1.12 (0.99–1.28)	0.081
Stage 2 hypertension	1.07 (0.90–1.26)	0.444
Taking anti-hypertensive	1.04 (0.92–1.19)	0.510
Chronic pain (Ref: No pain)		
Pain	**1.31 (** **1.18–1.44)**	**<0.001**
Not answered	1.06 (0.44–2.57)	0.894
Health Behaviors		
Smoking ≥100 cigarettes (Ref: <100)	**1.35 (** **1.23–1.48)**	**<0.001**
Binge drinking (No binge drinking)		
Regular binge drinking	1.04 (0.92–1.18)	0.542
Occasional binge drinking	**0.84 (0.75–0.94)**	**0.003**
Physical activity (Ref: Sometimes or often)		
Never or seldom	1.10 (0.94–1.27)	0.236
No answer or refused	1.27 (0.20–7.95)	0.799
Family physician visits (Ref: ≥1 visit in past 12 months)		
No physician visits	**0.85 (0.73–0.99)**	**0.039**
Anthropometric Measures		
BMI (Ref: Normal weight: 18.5–24.99)		
Underweight: <18.5	**1.87 (1.20–2.92)**	**0.006**
Overweight: 25–29.99	0.72 (0.48–1.07)	0.100
Obese: ≥30	0.63 (0.37–1.08)	0.091
Waist to hip categorical (Ref: Below cut–off; low risk)		
Above cut-off; High risk	1.11 (0.99–1.26)	0.073
Waist–to–height ratio (Below cut–off; low risk)		
Above cut-off; High risk	1.01 (0.88–1.15)	0.932
Disease risk (Ref: Least risk)		
Increased	1.09 (0.74–1.63)	0.659
High	1.17 (0.76–1.82)	0.478
Very high	1.09 (0.62–1.92)	0.765
Body fat percent (Ref: 0–26%)		
26%–31%	**1.32 (1.12–1.56)**	**0.001**
31%–36%	**1.28 (1.06–1.53)**	**0.009**
36%–41%	**1.79 (1.46–2.19)**	**<0.001**
41%–59%	**1.72 (1.36–2.18)**	**<0.001**
Dietary Intakes		
Average daily intakes of fiber sources (Ref: 0 to <1)		
≥1 to <2	1.02 (1.00–1.22)	0.061
≥2 to <3	**1.25 (1.09–1.44)**	**0.001**
≥3	**1.34 (1.06–1.70)**	**0.014**
Dietary Intakes */cont’d*		
Average daily intakes of pulses and nuts (Ref: 0 to <0.5)		
≥0.5 to <1	**0.84 (0.74–0.95)**	**0.004**
≥1 to <2	**0.89 (0.80–0.99)**	**0.040**
≥2	0.93 (0.77–1.12)	0.438
Average daily intakes of fat sources (Ref: 0 to <2.5)		
≥2.5 to <4	0.92 (0.79–0.08)	0.306
≥4 to <5	0.90 (0.75–1.08)	0.243
≥5	0.88 (0.73–1.07)	0.203
Average daily intakes of fish (Ref: No fish consumption)		
Consumes fish	1.08 (0.93–1.25)	0.317
Average daily intakes of omega-3 eggs (Ref: No omega–3 eggs)		
Consumes omega-3 eggs	0.91 (0.83–1.01)	0.076
Average daily intakes of fruits and vegetables (Ref: ≥6)		
0 to <2	**1.26 (1.04–1.52)**	**0.019**
≥2 to <3	**1.24 (1.05–1.46)**	**0.012**
≥3 to <4	1.04 (0.88–1.22)	0.666
≥4 to <6	1.13 (0.96–1.32)	0.142
Average daily intakes of pure fruit juice (Ref: No consumption)		
≤1	1.04 (0.94–1.15)	0.433
>1	1.28 (0.95–1.72)	0.101
Average daily intakes of salty snacks (Ref: No consumption)		
0 to ≤1	1.08 (0.95–1.23)	0.227
>1 day	1.23 (0.49–3.06)	0.663
Average daily intakes of calcium sources with high vitamin D content (Ref: ≥4)		
0 to <1	**0.73 (0.56–0.95)**	**0.018**
≥1 to <2	**0.73 (0.57–0.92** **)**	**0.009**
≥2 to <4	0.81 (0.65–1.01)	0.066
Average daily intakes of calcium sources with low vitamin D content (Ref: >0)		
No consumption	0.93 (0.82–1.05)	0.236
Average daily intakes of pastries (Ref: No consumption)		
>0 to ≤1	1.06 (0.91–1.23)	0.459
>1	**1.55 (1.12–1.15)**	**0.008**
Average weekly intakes of chocolate bars (Ref: No consumption)		
>0 to ≤0.6	1.01 (0.92–1.11)	0.850
>0.6	1.06 (0.86–1.32)	0.582

Note: *p*-values that are less than 0.05 level of significance are bolded.

## Supplementary Materials

The following are available online at https://www.mdpi.com/1660-4601/17/5/1493/s1, Table S1: Descriptions of Covariates.

## Disclaimer

The opinions expressed in this manuscript are the authors’ own and do not reflect the views of the Canadian Longitudinal Study on Aging. The AB SCREENTM II assessment tool is owned by Dr. Heather Keller. Use of the AB SCREENTM II assessment tool was made under license from the University of Guelph.
