# Supplementary material for: Nutritional Factors, Physical Health and Immigrant Status Are Associated with Anxiety Disorders among Middle-Aged and Older Adults: Findings from Baseline Data of The Canadian Longitudinal Study on Aging (CLSA)
Source: Int J Environ Res Public Health. 2020 Feb 26;17(5):1493. doi: 10.3390/ijerph17051493 (PMC7084187; doi:10.3390/ijerph17051493)
Supplement: Supplementary file 1 [file ijerph-17-01493-s001.pdf]

**Table S1: Descriptions of Covariates**	
1. Demographic, Social, and Economic Measures	
Sex	Men or women
Age	1) 45-55; 2) 56-65; 3) 66-75; and 4) 76-85 years
Education	1) < secondary school graduate; 2) high school graduate and/or with some post-secondary education; and 3) post-secondary degree/diploma; 4) non-response
Income	Estimated gross household income/past 12 months: 1) <$20,000; 2) $20,000- < $50,000; 3) $50,000- < $100,000; 4) $100,000-< $150,000; 5) ≥$150,000; 6) non-response
Relationship	1) single; 2) married/live with a partner/common-law; & 3) widowed/divorced/ separated
2. Physical Health Measures	
Multi-morbidity	Derived variable; chronic condition diagnoses summed: diabetes (includes borderline), heart disease or congestive heart failure, peripheral vascular disease or poor circulation in limbs, dementia or Alzheimer's disease, multiple sclerosis, epilepsy, migraine headaches, intestinal or stomach ulcers, bowel disorders (e.g., Crohn's disease, ulcerative colitis, irritable bowel syndrome), macular degeneration, mood disorder, back problems (excludes bromyalgia, arthritis), kidney disease/failure, rheumatoid arthritis, osteoarthritis (hands, hip and/or knee), or cancer. Categories: 1) no health condition; 2) one health condition; 3) two health conditions; 4) > two health conditions
Chronic pain	Derived from responses to two questions that asked if respondents were usually free from discomfort (yes/no) and number of activities that their pain/discomfort prevented (none, few, some, most). Coding: "free from pain" or "a few, some or most activities prevented by pain"
Blood pressure	American College of Cardiology Guidelines [1] and measured average blood pressure results: 1) normal (systolic < 120 mm Hg; diastolic < 80 mm Hg); 2) elevated hypertension (systolic 120-129 mm Hg; diastolic < 80 mm Hg); 3) stage one hypertension (systolic 130-139 mm Hg; diastolic 80-89 mm Hg); 4) stage two hypertension (systolic ≥ 140 mm Hg or diastolic ≥ 90 mm Hg); 5) currently taking anti-hypertensive medication
Smoking status	Two categories based on whether participants reported if they ever smoked at least 100 tobacco cigarettes in their lifetime [2]
Drinking behaviour	1) non-binge drinking; 2) occasional binge drinking (i.e., consumed alcoholic beverages < once/month but ≥ once/past 12 months); 3) regular binge drinking (i.e., men who had ≥ 5 drinks or women who had ≥ 4 drinks on one occasion ≥ once/month in the past 12 months) [3]
Physical activity	1) never/seldom; 2) sometimes/often; 3) non-response. Based on yes/no response to a question about engagement in light sports or recreational activities (e.g., bowling, golf with a cart, shuffleboard, badminton, fishing) in the previous seven days
3. Over-nutrition indicators	
Body mass index (BMI)	1) obese (≥ 30 kg/m^2); 2) underweight (< 18.5 kg/m^2); 3) healthy weight (18.5-24.99 kg/m^2); 4) overweight (25-29.99 kg/m^2).
Waist-to-hip ratio (WHR)	Based on the cut-off defining high risk (> 0.85 for female; >0.90 for male) [4].
Waist-to-height ratio (WHtR)	> 0.50 defines an increased risk [5]
Body fat percent	Based on dual-energy x-ray absorptiometry (DEXA): 1) <26%; 2) 26-31%; 3) 31-36%; 4) 36-41%; 5) 41- 59%
Disease risk	Based on BMI and WHR: 1) very high risk; 2) least risk; 3) increased risk;4) high risk [6]
4. Poor nutrition indicators	
Handgrip strength (HGS)	Based on Tracker Freedom wireless grip dynamometer results and categorized as under-nutrition (19.2 kgf (kilogram force) for women; 37.9 kgf for men between 45-64 years or 30.2 kgf for men; women 65 years$^+$), no under-nutrition, or not assessed [7]
Nutritional risk	Based on responses to the modified SCREEN© II instrument (Seniors in the Community: Risk Evaluation for Eating and Nutrition) and categorized as high risk (<38), low risk (≥38), and not assessed

Table S1: Descriptions of Covariates /cont'd..	
4. Poor nutrition indicators /cont'd..	
Sarcopenia	Based on the skeletal muscle index and screening algorithms [8]. Sex-specific quintile points were applied with the 20-percentile used as the cut-off to classify respondents as having sarcopenia or not [9]
Bone mineral densities	Measured by DEXA and categorized as osteoporosis (T-score ≤ -2.5), osteopenia (T-score -1 to -2.5), and normal bone density (T-score > -1) [10]
Iron deficiency anemia screen	Based on haemoglobin cut-off values of ≤ 119 g/L for women and ≤ 129 g/L for men [11]. For those who did not consent to blood work another category was included
5. Dietary intake measures [12]	
Fiber intake	High fibre breakfast cereals as well as whole wheat, bran, multigrain, and rye breads
Pulses & nuts	Legumes (beans, peas, lentils) and nuts, seeds and peanut butter
Fat sources	French fries or pan-fried potatoes, poutine; butter or regular margarine on bread or on cooked vegetables only; regular vinaigrettes, salad dressings, mayonnaise, homemade or commercial dips; beef, pork (ground, hamburgers, roast beef, steak, cubed); other meats (veal, lamb, game); patés, cretons, terrines; sauces and gravies (brown, white); sausages, hot dogs, ham, smoked meat, bacon; all egg dishes except omega 3 eggs (eggs, omelette, quiche); chicken, turkey
Fish<br>Omega-3 egg	0=no consumption, 1=at least consumed once
Fruits & vegetables	Fresh, frozen, or canned fruits, green salad, potatoes, carrots and other vegetables
Fruit juice	100% pure fruit juices (e.g. orange, grapefruit or tomato)
Calcium sources: high vitamin D content	Calcium-fortified milk (35% more calcium), whole and skimmed milk (3.25%, 2%, 1%, milk fat), low-fat and regular cheeses; milk-based desserts; calcium-fortified beverages and juices
Calcium sources: low vitamin D content	Yogurt (low-fat and regular) and calcium-fortified foods
Salty snacks	Regular chips, crackers
Pastries	Cakes, pies, doughnuts, pastries, cookies, muffins
Chocolate bars	Average weekly consumption

1. Whelton, P. K.; Carey, R. M.; Aronow, W. S.; Casey, D. E., Jr.; Collins, K. J.; Dennison Himmelfarb, C.; DePalma, S.M.; Gidding, S.; Jamerson, K.A.; Jones, D.W.; MacLaughlin, E.J.; Muntner, P.; Ovbiagele, B.; Smith, S.C; Jr.; Spencer, C.C.; Stafford, R.S.; Taler, S.J.; Thomas, R.J.; Williams, K.A. Sr.; Williamson, J.D.; Wright, J. T., Jr. 2017 ACC/AHA/AAPA/ABC/ACPM/AGS/APhA/ASH/ASPC/NMA/PCNA Guideline for the prevention, detection, evaluation, and management of high blood pressure in adults: A report of the American College of Cardiology/American Heart Association Task Force on Clinical Practice Guidelines. *J Am Coll Cardiol* **2018**, *71(19)*, e127-e248. doi: 10.1016/j.jacc.2017.11.006
2. Centers for Disease Control and Prevention. *National Health Interview Survey Glossary*. Available online: https://www.cdc.gov/nchs/nhis/tobacco/tobacco_glossary.htm. (accessed 11 January 2020)
3. Statistics Canada. (2016). Heavy Drinking. Available online: https://www150.statcan.gc.ca/n1/pub/82-625-x/2017001/article/54861-eng.htm (accessed 16 November 2018)
4. World Health Organization. *Waist circumference and waist-hip ratio: report of a WHO expert consultation, Geneva, 8-11 December 2008. Geneva: WHO*, 2011. Available online: https://www.who.int/nutrition/publications/obesity/WHO_report_waistcircumference_and_waisthip_ratio/en/ (accessed 11 January 2020)
5. Browning, L. M.; Hsieh, S. D.; Ashwell, M. A systematic review of waist-to-height ratio as a screening tool for the prediction of cardiovascular disease and diabetes: 0.5 could be a suitable global boundary value. *Nutr Res Rev* **2010**, *23(2)*, 247-269. doi: 10.1017/S0954422410000144

6. Douketis, J.D.; Paradis, G.; Keller, H.; Martineau, C. Canadian guidelines for body weight classification in adults: application in clinical practice to screen for overweight and obesity and to assess disease risk. *CMAJ* **2005**, *172(8)*, 995-998.
7. Guerra, R.; Fonseca, I.; Pichel, F.; Restivo, M.; Amaral, T. Handgrip strength cutoff values for undernutrition screening at hospital admission. *Eur J Clin Nutr* **2014**, *68(12)*, 1315.
8. Lourenço, R.A.; Pérez-Zepeda, M.; Gutiérrez-Robledo, L.; García-García, F.J.; Rodríguez Mañas, L. Performance of the European Working Group on Sarcopenia in Older People algorithm in screening older adults for muscle mass assessment. *Age Ageing* **2014**, *44(2)*, 334-338.
9. Yoshida, D.; Suzuki, T.; Shimada, H.; Park, H.; Makizako, H.; Doi, T.; Anan, Y.; Tsutsumimoto, K.; Uemura, K.; Ito, T. Using two different algorithms to determine the prevalence of sarcopenia. *Geriatr Gerontol Int* **2014**, *14*, 46-51.
10. National Osteoporosis Foundation. *Bone Density Exam/Testing*. Available online: https://www.nof.org/patients/diagnosis-information/bone-density-examtesting/. (accessed 11 January 2020)
11. World Health Organization. *Haemoglobin concentrations for the diagnosis of anaemia and assessment of severity. Vitamin and Mineral Nutrition Information System*. Geneva, World Health Organization, 2011 (WHO/NMH/NHD/MNM/11.1). Available online: http://www.who.int/vmnis/indicators/haemoglobin.pdf (accessed February 28, 2019)
12. Shatenstein, B.; Payette, H. Evaluation of the relative validity of the Short Diet Questionnaire for assessing usual consumption frequencies of selected nutrients and foods. *Nutrients* **2015**, *7(8)*, 6362-6374. doi: 10.3390/nu7085282
